# Programmatic and Communication Issues in Relation to Serious Adverse Events Following Ivermectin Treatment in areas Co-endemic for Onchocerciasis and Loiasis

**DOI:** 10.1186/1475-2883-2-S1-S10

**Published:** 2003-10-24

**Authors:** Nancy J Haselow, Julie Akame, Cyrille Evini, Serge Akongo

**Affiliations:** 1 Helen Keller International, B.P. 14227, Yaoundé, Cameroon; 2 Ministry of Public Health, Delegation for Center Province, Yaoundé, Cameroon

## Abstract

In areas co-endemic for loiasis and onchocerciasis, the classic Community-Directed Treatment using ivermectin (Mectizan^®^) must be adapted as additional program activities, better communication and tighter control of ivermectin stocks are required to minimize risk and manage serious adverse events following ivermectin treatment in patients co-infected with *Loa loa*. The importance of these serious adverse events on community participation in onchocerciasis control efforts has not been adequately studied. Program implementers do not as of yet fully understand the psychological impact of serious adverse events on communities and therefore have not designed communication strategies that adequately address the real concerns of community members. It is clear, however, that along with an effective case detection and management strategy, a reinforced communication strategy will be required to motivate at least 65% of the total population in onchocerciasis and loiasis co-endemic areas to participate in the treatment program and to take ivermectin over an extended period. This strategy must be based on research undertaken at the community level in order to address the concerns, fears and issues associated with adverse events due to ivermectin – to ensure that communities believe that the benefits of taking ivermectin outweigh the risks. In addition to an overall increase in the time required to sustain onchocerciasis control programs in co-endemic areas, each aspect of the reinforced program and communication strategy – rapid epidemiological assessments, materials development, training, advocacy, community sensitization and mobilization, case management and counselling, supervision, monitoring and evaluation will require additional resources and support from all stakeholders concerned.

## Review

### Introduction: How serious adverse events affect implementation of community-directed treatment with ivermectin

The issue of serious adverse events or side effects (SAEs) due to ivermectin in areas co-endemic for loiasis has been known for some time [1,2]. It was not until 1999, however, when the Center Province of Cameroon experienced a large number of cases with coma (23) and 3 deaths [3], that a more intensive effort was enlisted (i.e. Tours, France Meeting in October 1999 and 9^th ^Technical Consultative Committee (TCC9) meeting in March 2000) [4] to develop a plan to manage SAEs, including planning of an effective distribution strategy, more intensive supervision and surveillance of SAEs and more intensive and targeted information, education and communication (IEC) activities.

**Figure 1:**
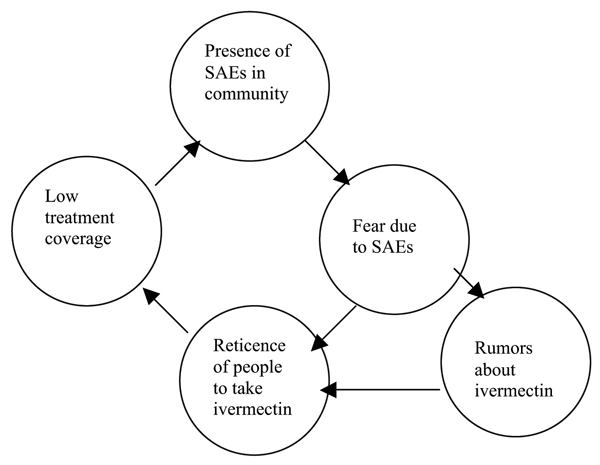
Vicious cycle of SAEs and low coverage caused by increased fear of taking ivermectin.

With better surveillance, other provinces of Cameroon and other countries (e.g. Sudan) are also reporting more SAE cases in areas co-endemic with loiasis and onchocerciasis. These co-endemic areas operate their programs with a unique challenge to manage all adverse side effects adequately, to maintain the cost per treatment at an acceptable level and to achieve and maintain a treatment coverage rate of at least 65% of the total population in order to adequately diminish transmission among the population and eliminate onchocerciasis as a public health problem and socio-economic importance [5-9]. This is not an easy undertaking. Because of the fear caused by serious adverse events [3,10-12], a reinforced strategy is required to motivate communities to participate in community-directed treatment with ivermectin (CDTI) and to take ivermectin. A review of several of the independent African Programme for Onchocerciasis Control (APOC) monitoring reports from 1999–2001 documented that mild and serious side effects were neither recorded nor reported in many projects, and in Cameroon, health education was not adequate to allay fears and misconceptions of community members about side effects. In the report of one of the CDTI Projects in the South West of Cameroon [12], the high rate of refusals (27.7%) among those eligible for treatment was closely linked to a high level of scepticism, doubt and pessimism among community members and the absence of a strong sensitization and mobilization effort. Likewise, an evaluation of the implementation of TCC9/Mectizan^® ^Expert Committee guidelines in areas of Cameroon co-endemic for onchocerciasis and loiasis undertaken by APOC and the TCC in October 2000 [11] found that, in general, communities did not have enough information on side effects to allay their fears. In the presence of SAEs, rumours and incomplete information, some community members acknowledged fear associated with even minor side effects and were understandably reticent to take ivermectin (Figure 1).

The presence of SAEs requires increased and improved health education and communication activities at the community and family level on early "warning" signs of SAEs, additional training and increased supervision at all levels, and additional program activities not necessary in areas where loiasis is absent [4,11]. The presence of SAEs not only increases the cost of implementing CDTI, but changes the very nature of the APOC CDTI strategy and in particular requires health personnel and communities to work together much more closely to manage the problems at the community level.

### Special issues to consider when implementing CDTI in the presence of Serious Adverse Events

Many articles reviewed [13-20] provide evidence on the critical importance of community participation in all aspects of CDTI – appropriate and targeted health education (in several cases to allay fear of side effects), community sensitization and mobilization to motivate participation, and the selection and training of community distributors of ivermectin (CDD) by the community for achieving and sustaining high treatment coverage levels. None of these articles, with the exception of TCC guidelines and Cameroon-based information [3,10,11,21-24], however, discussed the particular challenges faced by CDTI programs in co-endemic areas due to the presence of SAEs. As a result of the high number of SAE cases experienced in Cameroon during 1999, additional research was carried out in an attempt to better understand the issues related to SAE management. The Centre Pasteur of Cameroon study [21] further defined the parameters of SAEs (prevalence, timing of onset, risk factors), which allowed program recommendations to be made. They concluded that more training, more sensitization and more supervision are necessary to better manage SAE cases so that the prognosis on cases is improved. Using this information, evaluation findings [11] and lessons learned over 5 years of CDTI implementation in a loiasis and onchocerciasis co-endemic area of Cameroon, some of the special programmatic issues to consider when developing the distribution and communication strategy are outlined below. The strategy will necessarily be different between areas with SAEs due to ivermectin and those areas without this problem because of the medicalization of the CDTI program and the fear associated with presence of SAEs at the community level.

### Program-related issues in the presence of serious adverse events

#### Identification of onchocerciasis-endemic communities

In order to minimize risk of serious adverse events in loiasis-endemic areas, mass treatment with ivermectin is undertaken in only the onchocerciasis hyper-endemic and meso-endemic villages in accordance with the APOC's TCC guidelines. The villages are identified based on a rapid epidemiological assessment (REA) at the outset of the CDTI project.

Without adequate resources, careful planning, effective training or close supervision the categorization of villages can be inaccurate. Occasionally, some at-risk villages are excluded from the survey (and mass treatment) because they are inaccessible. It can also happen that hypo-endemic villages are included in mass treatment. Community sensitization prior to the survey is important to maximize participation [25] in order to give each eligible person an equal chance of inclusion in the survey. Since the survey is conducted by health personnel who do not always ensure the randomness of the sampling in each village, however, even with optimal community participation the representativeness of the results can be questionable. Adequate training for the REA therefore is an important element to ensure that only hyper- and meso-endemic villages are included in mass treatment in order to minimize unnecessary risk of SAEs to people living in onchocerciasis hypo-endemic areas.

It can happen that hypo-endemic villages are found close to meso-endemic villages, which can pose a problem during treatment, as hypo-endemic villagers do not often fully understand their exclusion and may seek treatment in neighbouring villages. Careful communication is needed to rectify these potential problems so that those people living in hypo-endemic villages, but who have onchocerciasis, seek and receive treatment at a referral hospital and not from a CDD. In addition, considering that the endemicity of onchocerciasis is not static due to environmental related issues, the REA results should be updated as the project evolves and adjustments made in order to truly eliminate onchocerciasis as a public health problem.

#### Planning

Planning and effective organization take on more importance when distribution needs to be more synchronized from village to village in order to better control the distribution of ivermectin to ensure proper surveillance of SAEs. So that all SAE cases receive adequate attention and care at each level, each district and each community must abide by their action plan once developed, which should include more frequent consultation meetings between communities and MOH personnel, supervised community selection of CDDs, updating of census figures, and a specific plan for SAE management, training, sensitization, distribution and supervision [23].

The decentralization of referral hospitals is important for the recovery of SAE cases, in part, because patients who are treated near their usual residence are more likely to be assisted by family members in their nursing care. These hospitals must have a well-trained team of professionals as well as necessary medication and equipment to properly manage SAE cases. Communities need to be informed of the designated referral hospital and be advised to go there as trained staff, medication and equipment to properly manage SAE cases are not necessarily found at the nearest hospital, which may be in an adjacent area not involved in CDTI.

A major challenge is to ensure that all communities in co-endemic areas where ivermectin is being distributed have access to a referral hospital as some communities are difficult to reach by road and may be more than 30 kms from a referral hospital. On the other hand, although cities are generally near a referral hospital, distributing ivermectin in cities with their more transient population and lack of cohesive community leadership, poses other problems in terms of selection of CDDs, taking an accurate census, implementing a motivating communication strategy to encourage participation in CDTI and surveillance of the population post-treatment.

#### Training

Early recognition of SAEs is one of the key elements in their effective management. SAE recognition, prevention, referral, counselling, management and reporting must be a primary focus of all training and retraining at all levels (CDDs, nurses, doctors) [26-28]. Ensuring a high level of CDD competence on recognition of early signs of SAEs is critical. Another important issue to address during training is that CDDs should provide community members with consistent, correct information on alcohol consumption before and after ivermectin treatment. Contradictions between information in manuals, health education messages and what the community believes to be true should be highlighted and clarified. IEC supports can be used as reminders on SAE information to help community distributors. In addition, because of a relatively high turn-over among government health staff, steps must be taken to ensure that any new staff who arrive during distribution receive adequate training.

In addition, a team of health personnel (at least 4 people) at each referral hospital must be well trained on diagnosis and management of cases so that all personnel who come in contact with an SAE case have the necessary background, understanding and motivation to manage the case properly. Interpersonal communication and counselling skills should also be taught so that hospital staff can give appropriate and caring feedback to family members [11]. The Ministry of Health and HKI in Cameroon have drafted a guidebook for the management of SAEs that can be reviewed and improved for wider use [29].

#### Selection of Community Distributors

Because of the increased knowledge and skills necessary to pass on the additional information to community members and to supervise post-treatment, the selection of the CDD is highly critical and should be supervised by health personnel after discussions with the community as to the type of person needed to carry out the duties. The CDD selected by the community must possess a minimal educational level, be stable, and be accepted by all in the community. This process is even more difficult in cities because of the lack of community cohesiveness and support.

#### Sensitization and Advocacy

Because SAE cases, and particularly poor management of these cases, cause fear and breed rumors [3,10-12], during the sensitization of communities an emphasis must be placed on ensuring that IEC materials and sensitization activities focus on identification and prompt referral of SAEs at the community level. Providing information on the reasons why side effects happen and their frequency and prognosis is also important in stopping rumors and allaying fears.

Counseling post-SAE may also serve to allay fears. The development of a counseling guide to be used by key community leaders to provide support to SAE cases and their families is underway, but will require additional training of key community members.

#### Distribution

The need to minimize problems associated with poor recognition and management of SAEs necessitates that individual communities have somewhat less control in the organization of mass treatment campaigns. A joint distribution strategy needs to be elaborated to better ensure that treatment and post-treatment are adequately supervised by health personnel in each village [4]. Activities must be synchronized in a health area so that health personnel are available and so that the ivermectin supply is controlled. Elements of the strategy might be:

◦ Villages are grouped for scheduled treatment in a logical order so that the nurse is available to supervise post-treatment in each village as per TCC guidelines.

◦ The CDDs, with community leaders, organizes and promotes the distribution time before hand and gives the ivermectin at a central site in the village to reach the greatest number of residents. Soon afterward, a mop-up can be done by the CDD.

◦ The CDD watches each person swallow the ivermectin and refuses to give any ivermectin to anyone for later consumption by other family members not physically present.

◦ The nurse collects all of the unused ivermectin within a day after the distribution is completed in the village. The control of ivermectin is critical in ensuring proper supervision of SAE cases. Training and monitoring tools are needed. Guidelines on the management of ivermectin at each level have been developed.

#### Identification, referral and care of SAE cases

Family members are usually the first to identify potential SAE cases based on the information that they are provided about potential adverse side effects due to ivermectin. They must be adequately informed to contact the CDD in their community, who in turn, must understand the importance of timely referral of mild cases to the nurses for treatment and immediate referral of serious cases to the closest designated referral hospital.

The cost to treat adverse effects is an issue with community members and a potential factor in low coverage as it can discourage participation. In Cameroon, mild adverse reactions to ivermectin are generally supported by the patient and family, whereas the referral hospital takes complete charge of all serious cases, paying directly for diagnostic tests, medication and hospitalization. Potential problems exist as those people with minor side effects often feel that they should be taken care of also. Additionally, since only confirmed cases are free, some patients delay going to the hospital because they are not sure if their adverse health is due to ivermectin or not – as they can not afford to pay for treatment if it turns out that their illness is unrelated to ivermectin. For patients from hypo-endemic areas, although the test for *loa loa *is required at the hospital, neither the test, the consultation nor the treatment is free. The cost information needs to be part of the IEC message to avoid false rumors or confusion that can discourage participation in CDTI [23].

#### Supervision and reporting

There is a need for increased and improved supervision by health personnel and CDDs in accordance with the TCC9 guidelines. In addition, follow-up by health personnel of recovering patients after hospitalization should be undertaken in communities [11,23]. Timely reporting and documentation must be strict. Unfortunately, the ability of many personnel to supervise and report accurately is often less than optimal due to the lack of skill, insufficient logistics and poor motivation. Additional training and support on how to effectively supervise is needed. Reporting procedures of potential and actual SAE cases must be discussed during training sessions as a critical element of proper SAE management so that each SAE case can be verified by project staff and follow-up undertaken to make sure hospitalized patients receive adequate care.

### Communication-related issues in the presence of serious adverse events

From the above issues cited in each element of the program plan and distribution strategy, it is clear that a reinforced, effective communication strategy including sensitization and mobilization, training, advocacy and health education – that is well designed and well implemented – is critical to long-term onchocerciasis control in the presence of SAEs. Many studies [30-37], in addition to those already referenced, provide evidence as to the importance of understanding community attitudes, beliefs and perceptions in order to design communication messages and materials that will better motivate participation in onchocerciasis control programs. Because most of these studies have taken place in areas without SAEs, they have not provided specific information related to community attitudes or behaviours in the presence of SAEs. Most of the communication and training strategies and materials developed have therefore focused on increasing knowledge about onchocerciasis and CDTI in order to promote compliance among communities for long-term ivermectin distribution. Methods to collect relevant knowledge, attitude and practice (KAP) data at the community level are well known, yet few KAP surveys have been done before strategy development in SAE areas. APOC has likewise developed a strategy [38] that, if correctly implemented, will greatly assist countries to put in place a more research-based, comprehensive advocacy and health education plan – to date the strategy has not been widely implemented.

Most of the available data on IEC issues related specifically to SAEs comes from Cameroon [3,10,11,22,24,39,40], where the majority of reported cases and fatalities due to ivermectin have occurred. In areas co-endemic with onchocerciasis and loiasis, information on adverse side effects has been included as part of health education and training efforts [26-28,40,41]. The community sensitization activities have typically been carried out by nurses and CDDs. How effective these efforts have been in transmitting the information and motivating behavior change is variable across projects based on coverage statistics, but it is clear that there is still considerable work to do before a comprehensive communication and training strategy is implemented that truly addresses the issues related to motivating full participation in CDTI in co-endemic areas.

In 1996, HKI conducted a KAP study in 4 health districts of Cameroon [40] from which a training guide [26] was developed that included a section on proper management of side effects. This guide was developed into a Regional Training Guide [27] for health personnel and CDDs that was adapted for use in Niger, Mali, Cameroon, Burkina Faso, Nigeria and Tanzania. After the large number of SAEs occurred in Okola in 1999, the Cameroon version was revised and additional information on SAE management [28] was added based on two community studies [3,22] which showed a general lack of information about CDTI and about SAEs. Key health education messages related to side effects were elaborated. A section on *How to convince people to take ivermectin *was included with responses to common concerns. Although the documents were well done, the messages do not seem to be adequate in light of the increased number of SAE cases. The training modules were well defined, yet at each level of the cascade training, the skills and knowledge do not seem to translate adequately into building community participation and motivating people to take ivermectin. These training modules have been continually revised based on feedback from users, but should be evaluated.

In December 1998 a workshop was held in Cameroon [41] to review messages and materials used by various projects and to decide on a uniform set of messages and materials for the National Onchocerciasis Control Program (NOCP). In one case, a KAP survey had been done to elaborate the messages and materials (posters, booklets, songs) based on the program objectives. In a few cases, information on the existence of SAEs was included as part of the project's IEC messages and materials. In the end, the standard set of messages and materials developed by the NOCP was not completely based on community level research and very little related to management of SAEs was included. None of the materials underwent testing before final production. In addition, a comprehensive communication strategy was not developed to accompany the agreed materials. Consequently, the messages have not been motivating in loiasis-endemic areas and the materials have not been well used by health personnel or CDDs. Likewise, in 1998 APOC developed a manual based in part on a multi-country study [20] that only touched on issues related to SAEs. Consequently some of the statements included in that manual [42], such as "the medicine is without danger" are not valid in co-endemic areas, and when told to community members can create doubt about the program.

Evaluation data [11,12], along with findings from a KAP survey [22] and three studies [3,10,21] done in co-endemic areas of Cameroon, have allowed some progress to be made in terms of defining a better, more targeted communication and training strategy. The most recent study [10] conducted by HKI at the end of the last 2001 CDTI campaign again reinforced the need for revised messages, more pertinent IEC materials based on community data, and the development and implementation of a more comprehensive communication strategy because the messages and strategy being used were not yet adequate to motivate at least 65% participation in CDTI (Table 1).

**Table 1:** Problems identified and recommendations made from studies to improve communication strategies in co-endemic areas

**Problems Identified**	**Recommendations made**
Absence of a global IEC strategy. [10,24]	Develop an overall communication strategy from community-based research. [10,24] The plan should include disseminating clear, consistent and complete tested messages to demystify SAEs, allay fears and motivate participation in CDTI via multiple, appropriate channels to reinforce messages from national to community level. [10,23]
Incomplete messages given in communities cause doubt and allowing rumours to continue [3,11,23] (i.e. SAEs emphasized without explaining cause and prognosis. CDDs don't inform people about potential side effects because they do not want to be seen as distributing a dangerous drug. [10]	SAE training, materials and messages include complete information – why side effects happen, how all medications can have side effects, what are particular side effects of ivermectin, how long side effects last, effectiveness of treatment of side effects, the system to take charge of side effects, the efficacy of ivermectin, and health problems associated with onchocerciasis. [3,10,11,21,23]
Mistrust of government enhances negative rumours related to SAEs and ivermectin. [23] Insufficient implication of administrative authorities in sensitisation activities. [24]	Have an official launching ceremony with health, traditional and administrative officials and media. [10,23] Conduct advocacy at the highest levels to reinforce messages given in the communities. [24]
Messages are not motivating to behaviour change – are not creating demand for ivermectin in communities and are not well enough crafted to vanquish fear of SAEs. [10,11] There is insufficient community involvement in developing the messages. [24]	Design messages based on KAP survey done in and with communities. [10,24] Message must appeal to the population at the physical, emotional and practical level so the risk and fear of SAEs is less important than the treatment for onchocerciasis. [23,38] Evaluate the communication strategy to know if the messages were motivating enough to change behavior. Apply lessons learned to continually improve the strategy and messages. Encourage the use of testimonials of villagers. Monitor villages to know if the messages are being heard and understood. [10]
Health personnel are not reinforcing CDD messages adequately: source is important on medical issues like SAEs. [10,11,23]	Systematize sensitization activities by nurses before, during and after the distribution. [10,22,23]
There is insufficient competence in communication techniques at all levels. [10,23,24]	Include practicum on communication techniques in nurse and CDD training. [10,23,24] Encourage a positive, caring attitude among CDDs and health personnel toward SAE cases. [11] Reinforce skills during supervision. [23]
Visual supports are not well understood without explanation, nor are they often used to give health education. [10,22] There are insufficient IEC materials related to SAEs for health professionals and communities. [11,22,24]	Develop IEC supports that motivate, are graphic and self-explanatory based on information from community research. Explain the content of supports during health education sessions. [10] Produce enough materials for wide distribution in villages. Finalize supports specifically for health professionals on SAE management. [10,11,22]
Communities are not adequately informed or implicated in management of SAEs. There is a lack of information at the village level. [3,10-12,21-24]	Train CDDs and community leaders on SAEs, detection and referral. [11] Train church, school and social leaders to help sensitize the community and counsel SAE cases. [10,11,23] Train medical staff to counsel recovering SAE cases. [11] Sensitize communities before, during, and after distribution, including improved health education of families to understand the signs of alarm for SAEs and to know what to do about them. [3,11,21,23]
Absence of advocacy materials. [24]	Develop an advocacy kit to target resources. [24]
Insufficient number of IEC experts. [24]	Identify experts outside Onchocerciasis control program that could be tapped. [24]
Insufficient data on the relationship between SAEs and coverage. [11,23,24]	Conduct well-designed study to assess the relative importance of SAEs to coverage levels. [11,23,24]

### Conclusions and Research Recommendations

**Figure 2:**
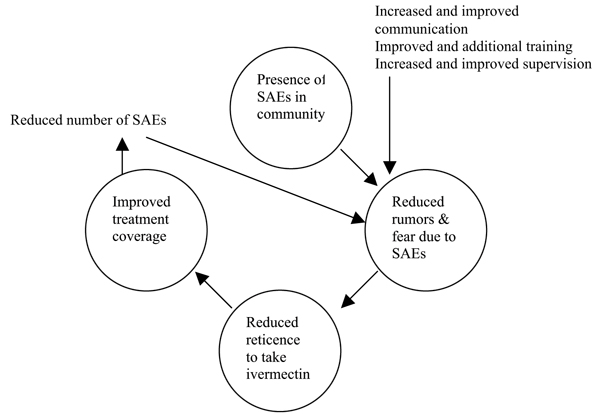
Ending the vicious cycle of SAEs and low coverage caused by increased fear of taking ivermectin.

It is clear that the presence of SAEs, even when they are well managed, requires a reinforced approach to convince people that the benefits of taking ivermectin outweighs the risk. Increased and improved supervision, training, and communication activities and skills are all needed to allay the fears caused by actual side effects and by rumours. Reducing fears will help to increase treatment coverage, which will in turn reduce the number of SAEs over time, which will increase participation in CDTI so that onchocerciasis can be eliminated as a public health problem (Figure 2).

So what is needed to more adequately address the program and communication issues related to SAEs and to better ensure the success of CDTI in co-endemic areas?

A study could be conducted to look at the reason why the majority of reported cases and fatalities due to ivermectin have occurred in Center Province of Cameroon. This phenomenon is unclear and should be explored in further depth. Is it due to biological or environmental factors, an ineffective management of SAE cases as compared to other countries or perhaps better surveillance and reporting in Cameroon?

An operational research study should be conducted in co-endemic areas to find out the comparative weight of various factors on coverage, including the general lack of information about CDTI, the lack of information specifically about SAEs, fear caused by the presence of SAEs, the motivation of CDDs, ineffective SAE management, the cost of SAE management to the family, and lack of concern for onchocerciasis as an important personal or community health problem, among others factors. Taking ivermectin at the population-level could be analyzed as a cost-benefit issue to gain insight on the difficulty of full participation over time. Furthermore, evaluations of CDTI projects in co-endemic areas should be conducted to assess and compare the additional costs associated with implementing CDTI in co-endemic areas and the additional time that it will take before program activities can be sustainable.

The existing training modules and actual training sessions should be reviewed and revised as necessary based on a thorough evaluation of their success in teaching skills and motivating effective action at all levels. This could be conducted by an expert trainer who could then make recommendations for improvement and design an intensive training-of-trainers skills workshop, (if warranted), to enhance the transfer of information from one level to the next.

Finally, well-designed qualitative research should be conducted in communities affected by SAEs to identify their real concerns, attitudes, beliefs, perceptions and practices related to participation in CDTI. These findings can by used to develop a comprehensive communication strategy, coupled with the effective management of side effects. The research can be used to design and test messages that will motivate community members to believe that the benefits of taking ivermectin are greater than the risks associated with SAEs – that onchocerciasis is a serious disease and its elimination is worth a long-term community effort, that ivermectin is effective, that adverse side effects can be recognized and effectively treated, that the cost is not too high to the family, that no one needs to die or be permanently disabled from taking ivermectin, and that no one needlessly go blind because of fear of SAEs.

## List of abbreviations

APOC African Program for Onchocerciasis Control

CDD Community-Directed Distributor

CDTI Community-Directed Treatment with Ivermectin

HKI Helen Keller International

IEC Information, Education and Communication

NOCP National Onchocerciasis Control Program

REA Rapid Epidemiological Assessment

SAE Serious Adverse Effect or Serious Adverse Event

TCC Technical Consultative Committee

## Competing interests

The Mectizan^® ^Donation Program, who sponsored the author's participation in the Scientific Working Group meeting, donates Mectizan^® ^to the HKI-supported CDTI projects.

APOC, who requested the authors to write this paper on their behalf for the meeting, provides program funding to HKI/Cameroon-supported CDTI projects.

## Authors' contributions

NJH drafted and finalized the paper and presented it at the Scientific Working Group on SAEs in Loa-endemic Areas, Manchester 28–30 May 2002.

JA, CE, and AS provided ideas and inputs via team discussions and edited the draft paper before finalization.
